# EFFECT OF TIME-RESTRICTED EATING ON NEUROVASCULAR COUPLING AND METABOLIC PROFILE IN OLDER ADULTS

**DOI:** 10.1093/geroni/igae098.3956

**Published:** 2024-12-31

**Authors:** Ana Clara Costa Pinaffi da Langley, Camila Bonin Pinto, Keith Kleszynski, Stefano Tarantini, Andriy Yabluchanskiy

**Affiliations:** University of Oklahoma Health Sciences, Oklahoma City, Oklahoma, United States; University of Oklahoma Health Sciences, Oklahoma City, Oklahoma, United States; University of Oklahoma Health Sciences Center, Oklahoma City, Oklahoma, United States; University of Oklahoma Health Sciences, Oklahoma City, Oklahoma, United States; University of Oklahoma Health Sciences, Oklahoma City, Oklahoma, United States

## Abstract

Vascular cognitive impairment and dementia (VCID) account for up to 30% of dementia cases worldwide, representing an important public health challenge. Normal cognitive function requires adequate cerebral blood flow (CBF) regulation. Therapeutical strategies aimed at rescuing age-related CBF dysregulation can delay or mitigate the incidence of VCID. Time-restricted eating (TRE) is an accessible alternative to caloric restriction that has demonstrated benefits on cardiometabolic outcomes. However, the effect of TRE on cerebrovascular function in older adults remains unexplored. Therefore, this pilot study aimed to investigate whether TRE improved cerebrovascular function and metabolic profile in heathy older adults. Participants were instructed to maintain a 10:14 intermittent fasting regimen daily for 6 months. Outcomes of interest were changes from baseline to endpoint in neurovascular coupling (NVC) response assessed with near-infrared spectroscopy and metabolic profile assessed using metabolomics analysis. Six participants (100% female, mean age: 59±5 years) completed the pilot study. Six months of TRE were associated with a significant increase in NVC responses (n=5) in the dorso-lateral prefrontal cortex, the region relevant to the cognitive stimulation paradigm. TRE was also associated with an increase in the levels of tetrahydrofolate, L-arginine and nicotinamide, metabolites involved in vascular health (n=5). TRE resulted in an increase in several metabolites directly implicated in antioxidant, anti-inflammatory, or anti-senescence pathways in the vasculature. Further, principal component analysis revealed a distinct clustering of the before and after TRE data. Our limited dataset indicates that TRE may improve NVC and the availability of metabolites relevant to vascular health in older adults.

